# Short-term outcome and differences between rural and urban trauma patients treated by mobile intensive care units in Northern Finland: a retrospective analysis

**DOI:** 10.1186/s13049-015-0175-2

**Published:** 2015-11-05

**Authors:** Lasse Raatiniemi, Janne Liisanantti, Suvi Niemi, Heini Nal, Pasi Ohtonen, Harri Antikainen, Matti Martikainen, Seppo Alahuhta

**Affiliations:** Department of Anaesthesia and Intensive Care, Lapland Central Hospital, Rovaniemi, Finland; Centre for Pre-Hospital Emergency Services, Oulu University Hospital, Oulu, Finland; Division of Intensive Care Medicine, Oulu University Hospital, Oulu, Finland; Faculty of Medicine, University of Oulu, Oulu, Finland; Division of Operative Care, Oulu University Hospital, Oulu, Finland; Medical Research Center, University of Oulu and Oulu University Hospital, Oulu, Finland; Department of Geography, University of Oulu, Oulu, Finland

**Keywords:** Emergency medical services, Helicopter, Rural, Urban, Finland

## Abstract

**Background:**

Emergency medical services are an important part of trauma care, but data comparing urban and rural areas is needed. We compared 30-day mortality and length of intensive care unit (ICU) stay for trauma patients injured in rural and urban municipalities and collected basic data on trauma care in Northern Finland.

**Methods:**

We examined data from all trauma patients treated by the Finnish Helicopter Emergency Medical Services in 2012 and 2013. Only patients surviving to hospital were included in the analysis but all pre-hospital deaths were recorded. All data was retrieved from the national Helicopter Emergency Medical Services database, medical records, and the Finnish Causes of Death Registry. Patients were defined as urban or rural depending on the type of municipality where the injury occurred.

**Results:**

A total of 472 patients were included. Age and Injury Severity Score did not differ between rural and urban patients. The pre-hospital time intervals and distances to trauma centers were longer for rural patients and a larger proportion of urban patients had intentional injuries (23.5 % vs. 9.3 %, *P* <0.001). The 30-day mortality for severely injured patients (Injury Severity Score >15) was 23.9 % in urban and 13.3 % in rural municipalities. In the multivariate regression analysis the odds ratio (OR) for 30-day mortality was 2.8 (95 % confidence interval 1.0 to 7.9, *P* = 0.05) in urban municipalities. There was no difference in the length of ICU stay or scores. Twenty patients died on scene or during transportation and 56 missions were aborted because of pre-hospital death.

**Conclusions:**

The severely injured urban trauma patients had a trend toward higher 30-day mortality compared with patients injured in rural areas but the length of ICU stay was similar. However, more pre-hospital deaths occurred in rural municipalities. The time before mobile ICU arrival appears to be critical for trauma patients’ survival, especially in rural areas.

## Background

Accidents are the most common cause of death for persons aged 1–44 years in Finland, with 48.8 deaths per 100,000 inhabitants in 2012 [1]. Trauma systems have been shown to reduce mortality and improve long-term outcomes [2, 3] but early recognition and rapid transportation of seriously injured patients directly to trauma centers are crucial factors in pre-hospital trauma care [4, 5]. Trauma is the next most common cause of dispatch of helicopter emergency medical services (HEMS) in Scandinavia [6]. Several studies have shown that HEMS have a positive impact on trauma outcomes [7–9], whereas others have not been able to document a benefit [10–12].

Rural areas have higher injury-related mortality rates than urban areas [13–16] and pre-hospital deaths appear to be more common in rural areas [13, 15, 16]. However, the influence of short pre-hospital response time or transport time to trauma center on mortality is undetermined [17]. Distances to definitive care in geographically large Northern Finland can be very long and it is unknown whether outcomes differ between patients injured in rural vs. urban locations.

Our aim was to compare the 30-day mortality rate (primary outcome measure) and length of intensive care unit stay (secondary outcome measure) of rural and urban trauma patients. We also aimed to describe the incidence, demographics, and critical care of trauma patients treated by the Finnish HEMS (FinnHEMS) units in Northern Finland.

## Methods

This was a retrospective, observational study of a 2-year cohort from two mobile ICU units operated by FinnHEMS. The study protocol was accepted by Statistics Finland, and statements were obtained from the local ethics committee of the Northern Ostrobothnia Hospital District as well as central hospitals following the local protocol.

### Emergency medical and trauma system in Northern Finland

The primary study area was the five northernmost hospital districts in Northern Finland, which had 741 135 inhabitants in 2012, covering approximately 50 % of the surface area of Finland. EMS consists of first responders and basic and advanced level ambulances. HEMS are provided by nation-wide FinnHEMS. In Northern Finland, a physician-staffed unit (FinnHEMS 50) operates out of Oulu University Hospital and a paramedic-staffed unit (FinnHEMS 51) operates out of Rovaniemi airport. A total of 464 000 inhabitants can be reached within 30 min and a helicopter or rapid response car is used, depending on operational factors. Annually, FinnHEMS 50 and 51 units, together, care for 800–1000 patients. Four central hospitals and one university hospital are located in the area.

The FinnHEMS is dispatched criteria-based for all life-threatening situations and high-energy accidents simultaneously with ground ambulances, from an emergency communication center [18]. Specially trained paramedics in FinnHEMS 51 may intubate an unconscious patient using ketamine and sedatives with strict systemic operative procedures, but online consultation with a pre-hospital anesthesiologist is always required. Inter-hospital transfers of critically ill patients are performed by ground ambulances and, if needed, supplemented by hospital staff.

### Patients

All patients treated by FinnHEMS and recorded as trauma patients (blunt or penetrating) in the electronic HEMS database from 1 January 2012 to 31 December 2013 were identified. Pre-hospital deaths (cancelled missions because of pre-hospital death or those declared dead by FinnHEMS) were also retrieved but not included in the analysis because patients’ identification is not routinely recorded in the database if the mission is aborted. Pre-hospital data (time intervals, pre-hospital therapy, transport method, escorted by FinnHEMS, the use of a helicopter) were retrieved from the HEMS database and coupled to hospital data using personal identification numbers. The hospital data (key emergency therapy defined by Utstein style reporting for major trauma [19], location where the patient was discharged, and Pre-Injury American Society of Anesthesiologists Physical Status [ASA-PS] classification system) were retrieved from the medical records. Data on intensive care (Sepsis-Related Organ Failure Score at admission and the maximum score; the Acute Physiology and Chronic Health Evaluation-II score; Simplified Acute Physiology Score; Therapeutic Intervention Scoring System score; and length of respiratory therapy) were obtained from the databases of the intensive care clinical information systems of each hospital. The Injury Severity Score (ISS) of the patients who survived to hospital was calculated by the main researcher (LR), who is certified in the use of the Abbreviated Injury Score [20]. Thirty-day mortality data was retrieved from the Causes of Death Registry maintained by Statistics Finland.

The road and straight-line (Euclidean) distances from the site of injury to the helicopter base and referral hospital were calculated using ArcGIS 10.2 software (ESRI, Redlands, CA). The statistical grouping of municipalities by Statistics Finland was used to identify urban and rural patients [21]. This classification groups municipalities into three categories (urban, semi-urban, and rural) according to the proportion of inhabitants living in urban settlements and the population of the largest settlement. In this study, the municipalities were divided into two categories, urban and rural, with the latter including semi-urban municipalities.

Response time was defined as the time from dispatch to arrival on scene. Transport time was defined as the time between the start of transport and the patient’s arrival at the hospital. On-scene time was defined as the time between FinnHEMS arrival on scene and the start of transport. Injury was defined as severe if the ISS score was >15.

### Statistical analysis

Data are expressed as medians with 25^th^–75^th^ percentiles and *P* <0.05 was considered significant. The Mann–Whitney test was used to compare continuous data between the groups, and Pearson’s chi-square test was used to compare categorical variables. A multivariate logistic regression model was built using a maximum of two adjusting covariates at a time to assess the impact of the type of municipality on 30-day mortality. The number of adjusting covariates was based on a relatively low number of trauma deaths. The adjusting covariates used were age, gender, ISS score, the type of HEMS unit, airway distance to the site of injury and the following HEMS time intervals: response, on-scene and transport times. Age and ISS score were categorized, since the linearity assumption did not hold. Only the severely injured patients were included in the analysis because no deaths occurred when the ISS score was less than sixteen. The results of the model with the lowest log-likelihood function are presented.

Data were analyzed using IBM SPSS Statistics for Windows, Version 22.0 (IBM Corp., Armonk, NY). We did not perform a power calculation because of the retrospective nature of the study and because the HEMS database had data only from the past two years (2012 and 2013).

## Results

### Patients and injuries

**Fig. 1:**
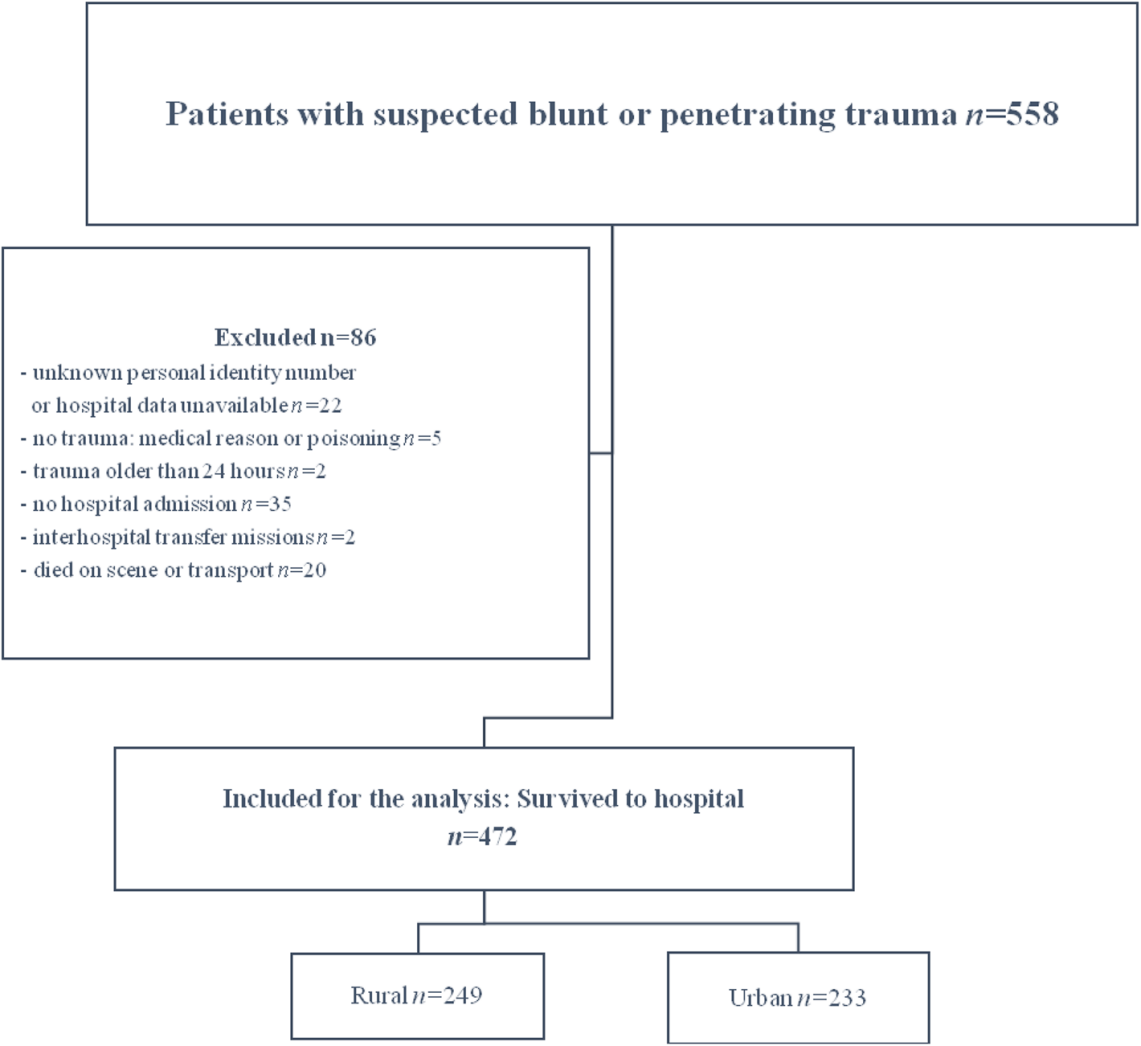
Flow chart of the study participants

A total of 558 trauma patients were treated by FinnHEMS during the study period, accounting for 29.3 % (558/1904) of all patients examined. Twenty trauma patients (3.6 %) died in the pre-hospital setting. We included a final total of 472 patients in the analysis (Fig. 1). The characteristics of the patients who survived to hospital are presented in Table 1. Injuries were classified as severe in 33.1 % (156/472) of cases with most patients suffering blunt injuries. We found no significant differences between the rural and urban groups regarding age, sex, ISS score, or ASA-PS scores (Table 1). Traffic accidents were the most common type of unintentional injuries, and intentional injuries were more common in urban municipalities.

**Table 1:** Characteristics of the patients who survived to hospital

Variable	Missing data	All *n =* 472	Rural *n =* 249	Urban *n =* 223	*P*-value
Age, median	0	33 (20–55)	39 (20.5–58)	28 (19–52)	0.28
Male	0	330 (69.9)	182 (73.1)	148 (66.4)	0.11
ISS, median	0	9 (3–17)	9 (4–17)	9 (2–17)	0.42
ISS > 15	0	156/472 (33.1)	85/249 (34.1)	71/223 (31.8)	0.60
AIS Head > 3	0	66/472 (14)	39/249 (15.7)	27/223 (12.1)	0.27
AIS Abdomen > 3	0	7/472 (1.5)	2/249 (0.8)	5/223 (2.2)	0.26
ASA-PS I-II	0	436/472 (92.4)	230/249 (92.4)	206/223 (92.4)	0.82
III-IV	0	36/472 (7.6)	19/249 (7.6)	17/223 (7.6)	
Accidents (all)	0	390/472 (82.6)	224/249 (90)	166/223 (74.4)	<0.001
Traffic	0	214/390 (54.9)	105/224 (46.9)	109/166 (65.7)	<0.001
All-terrain vehicle/snowmobile	0	31/390 (7.9)	27/224 (12.1)	4/166 (2.4)	<0.001
Falls	0	108/390 (27.7)	66/224 (29.5)	42/166 (25.3)	0.36
Machinery or hit by blunt object	0	27/390 (6.9)	17/224 (7.6)	10/166 (6)	0.55
Intentional injuries (suicidal or assaults)	0	74/472 (15.9)	23/249 (9.3)	51/223 (23.5)	<0.001
Suicidal	0	31/472 (6.6)	11/249 (4.4)	20/223 (9)	0.46
Assaults	0	43/472 (9.1)	12/249 (4.8)	31/223 (13.9)	<0.001
Dominant type of injury					
Blunt	0	408/472 (86.4)	220/249 (88.4)	188/223 (84.3)	0.20
Penetrating	0	64/472 (13.6)	29/249 (11.6)	35/223 (15.7)	0.20

Values are presented as number (%) or median (25^th^–75^th^ percentiles)

### Distances, pre-hospital times, and transportation of trauma patients

**Fig. 2:**
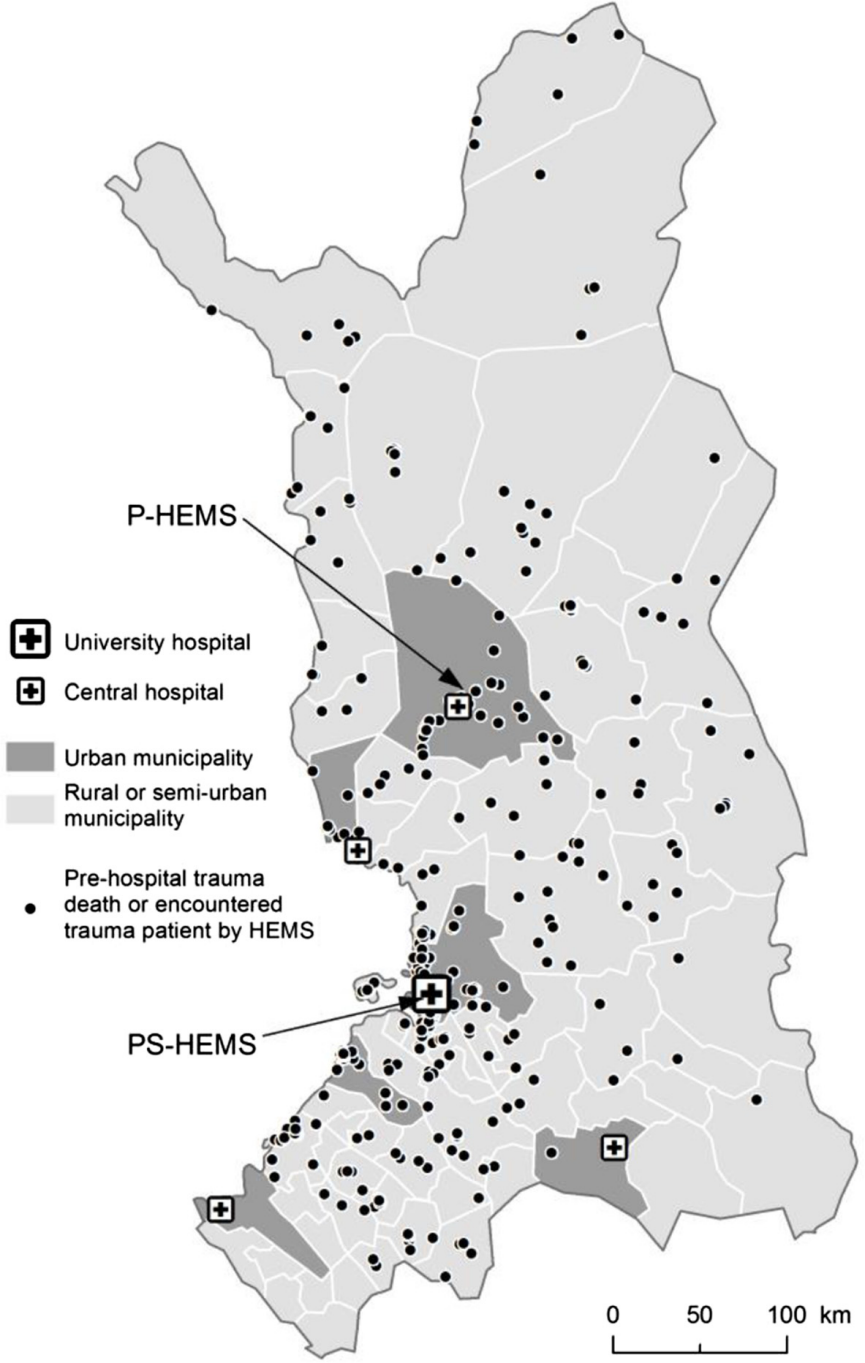
Geographical locations of trauma patients encountered by hospital emergency medical services (HEMS) and sites of pre-hospital trauma death

Response and on-scene times, as well as median distances from the site of injury to the referral hospital, were significantly longer in the rural group than the urban group (Table 2). Of the severely injured patients, 48.7 % (76/156) were injured more than 50 km from the helicopter base. The locations of the sites of injuries are illustrated in Fig. 2. The helicopter was used to respond to 52.6 % (246/472) of the patients, more often in rural municipalities (80.7 % vs. 20.2 %, *P* <0.001). Helicopter transportation was used for 21.2 % (100/472) of the patients. Of the severely injured patients, 21.2 % (33/156) were transported by helicopter and 88.5 % (138/156) were escorted by HEMS providers. Direct transportation from the site of injury to the trauma center at the university hospital was used for 78.8 % (123/156) of the severely injured patients, whereas 12.2 % (19/156) were first admitted to the central hospitals and later transported to the university hospital. The remaining 9.0 (14/156) were admitted only to central hospitals.

**Table 2:** Time intervals and distances

Variable	Missing data	All *n =* 472	Missing data	Rural *n =* 249	Missing data	Urban *n =* 223	*P*-value
Airway distance from scene to the receiving hospital (km)	21	45.3 (5.9–117.4)	12	112 (67.1–143.9)	9	5.7 (2.6–20.3)	<0.001
Road distance from scene to the receiving hospital (km)	21	55.4 (8.9–144.1)	12	137.7 (82.1–172.7)	9	8.1 (3.8–23.9)	<0.001
Transport time from scene to receiving hospital (min)	146	33 (12–51)	67	46.5 (35.8–64.2)	79	11 (7–21.75)	<0.001
Time from HEMS dispatch to the HEMS arrival on scene (min)	0	24 (11–43)	0	39 (28–53)	0	11 (8–18)	<0.001
On scene time (min)	95	18 (10–31)	42	22 (11–35)	53	15 (9–24)	<0.001
Airway distance from the HEMS base to scene (km)	67	36.9 (7.2–107)	44	102.1 (64.9–130.2)	23	7.2 (3.1–17.1)	<0.001
Road distance from the HEMS base to scene (km)	67	51.6 (8.7–131.8)	44	127.8 (78.6–163.9)	23	8.7 (4.6–20)	<0.001

Values are presented as median (25^th^–75^th^ percentiles)

### Critical care

Pre-hospital endotracheal intubation or a supraglottic airway device was used for 13.3 % (63/472) of patients, with no difference between the rural and urban groups (14.0 % vs. 12.6 %, *P* = 0.63). Key emergency interventions were performed for 15.3 % (72/472) of the patients, with no difference between the rural and urban groups (14.9 % vs. 15.7 %, *P* = 0.80). The most common procedures were neurosurgical (68.1 %, 49/72) and laparotomies, interventional radiological procedures, and thoracotomies were performed five times each. A total of 40.3 % (190/472) of patients were admitted to the ICU and we found no difference between the groups for length of ICU stay, respiratory therapy, and ICU severity scores (Table 3).

**Table 3:** Length of intensive care unit (ICU) stay and respiratory therapy, ICU mortality, and ICU scores

Variable	Missing data	All *n =* 190	Rural *n =* 105	Urban *n =* 85	*P*-value
Admission in ICU	0	190/472 (40.3)	105/249 (42.2)	85/223 (38.1)	0.37
Length of ICU stay (days)	18	1.8 (0.9–4.9)	1.8 (0.9–5.2)	1.8 (0.9–5.3)	0.53
Respiratory therapy during ICU stay	0	88/190 (46.3)	47/105 (44.8)	41/85 (48.2)	0.89
Length of respiratory therapy (hh:mm) *n = 88*	0	34:23 (6:00–115:19)	36:27 (7:11–115:12)	32:00 (4:00–95:00)	0.62
TISS total	25	77 (40–168)	84 (43.5–171)	70 (37.3–160.5)	0.53
SAPS	30	24 (17–40)	25.5 (17–40)	24 (17–38.5)	0.67
APACHE II	31	13 (8–20)	13 (8–19.5)	12.5 (7–20)	0.69
SOFA at admission	25	4 (2–7)	4 (2–7)	4 (2–7)	0.54
SOFA max	21	5 (2.5–9)	5 (2–9.8)	5 (3–9)	0.89

Values are presented as number (%) or median (25^th^–75^th^ percentiles)

### Outcomes

Overall 30-day mortality for patients who survived to hospital was 6 % (28/469; lost-to follow-up *n* = 3; 95 % confidence interval, 3.9–8.2 %) and 18.2 % (28/154; lost to follow-up *n* = 2; 95 % confidence interval, 12.1–24.3 %) for the severely injured patients. All who died within 30 days had an ISS score > 15. Four (0.8 %) patients died either in the emergency room or during an emergency operation and urban, severely injured patients tended to have higher 30-day mortality (23.9 % vs. 13.3 %, *P* = 0.09). In the best logistic regression model for 30-day mortality, the ISS score and age were adjusting covariates. In this model, the odds ratio (OR) for the urban municipality was 2.8 (95 % confidence interval 1.0 to 7.9, *P* = 0.05). The majority of patients were discharged home after primary admission to central or university hospitals, with no difference between the rural and urban groups (Table 4).

**Table 4:** Patient outcomes

Variable	Missing data	All *n =* 472	Rural *n =* 249	Urban *n =* 223	*P*-value
30-day mortality, all patients	3	28/469 (6.0)	11/246 (4.5)	17/223 (7.6)	0.15
30-day mortality, ISS>15	2	28/154 (18.2)	11/83 (13.3)	17/71 (23.9)	0.09
Proportion of deaths resulting from blunt injury	0	26/28 (92.9)	11/11 (100)	15/17 (88.2)	0.24
Proportion of deaths resulting from accidental injuries	0	23/28 (82.1)	10/11 (90.9)	13/17 (76.5)	0.33
Discharged to home	0	301/472 (63.8)	149/249 (59.8)	152/223 (68.2)	0.60
Discharged to home ISS>15	0	44/156 (28.2)	21/85 (24.7)	23/71 (32.4)	0.29

Values are presented as number (%)

A total of 19 patients died on scene and life-saving therapy was attempted for only three; one died during transportation to the hospital. Of the patients who died in a pre-hospital setting, 20 % (4/20) were injured in rural municipalities. A total of 56 missions were aborted before FinnHEMS arrival because death was declared by other units. Of the aborted missions, 73.2 % (41/56) were in rural municipalities. The number of deaths per aborted mission is unknown.

Of the patients transported directly to the university hospital by passing the nearest central hospital (*n* = 43), two patients died within 30 days. One patient of the 29 patients transferred from a central hospital died within 30 days.

## Discussion

This study found that despite longer distances and prolonged transport times from rural scenes, with comparable patient populations, urban trauma patients had a trend to higher 30-day mortality compared with rural patients. Delays in pre-hospital care providers discovering and arriving at the scene have been considered detrimental to outcomes in severely injured patients [22]. As a result, rural trauma patients enter the medical system with more advanced pathophysiological derangements. Prolonged time to definitive care has been thought to be associated with continued physiological abnormalities and increased mortality, which may not occur in an urban setting with a short total pre-hospital time [23]. Our findings do not support this concept. The higher mortality among urban patients in the present study may be explained by a selection bias. Early mortality, i.e. deaths declared by other units before FinnHEMS arrival resulting in abortion of the mission, was significantly higher in rural settings. A recent systematic review suggests that for undifferentiated trauma patients, shorter response time may have a positive influence on mortality [17]. The urban patients in our study survived to hospital more often because of shorter pre-hospital times, while a larger proportion of the major trauma rural patients died before accessing advanced emergency medical care.

Several groups have investigated differences in mortality between rural and urban trauma patients. McGuffie et al. and McCowan et al. found no differences in mortality between urban and rural patients [24, 25]; however, because of inconsistencies in definitions and methodology, comparisons with our study are difficult. Three papers have described the management of trauma patients by air medical transportation in remote and very remote areas in Western Australia. The mortality rate in very remote areas was four times the rate of that for a major city [15] and the authors found that remoteness is more important than distance regarding the risk of death [26]. Another study by the same group reported equivalent mortality in a major city and rural areas if the patients survived to the tertiary hospital [27]. However, these studies included only major trauma patients (ISS > 15); trauma patients who died before transfer were excluded, and the setting was unique geographic isolation that required prolonged transport times. Similar to our observations, a Norwegian study investigating inter-hospital transfer of trauma patients to a trauma center showed lower mortality for patients transferred the longest distance [28].

The number of pre-hospital deaths and the deaths before accessing FinnHEMS was considerably higher in rural environments. A high proportion of pre-hospital deaths, especially in rural areas, has been reported by several authors [13, 15, 16] and time from trauma to first-provider input is a significant predictor of the risk of death [22, 27].

Despite longer pre-hospital times, rural patients did not have longer or more complicated ICU stays than urban patients. More than half of the severe injuries occurred in rural municipalities and time to advanced therapy such as pre-hospital intubation may have been longer without FinnHEMS units. The large proportion of rural patients highlights the importance of HEMS in large geographical areas with only one university-level trauma center. Definitive care in trauma centers for rural patients can seldom be achieved within the “golden hour” even with helicopter transportation. However, evidence supporting the “golden hour” theory in trauma is inconclusive [17, 29].

Thirty-day mortality in the present study was similar to a Scandinavian HEMS study [30], but differences in dispatch criteria, severity of injuries, and pre-hospital response times, make comparisons with our study difficult. A focus on trauma care has the potential to save life-years, as roughly one-third of severely injured patients were discharged to home after primary admission in our study. The patients were also relatively young and the majority had only minor comorbidities.

In our study, less than two severely injured or deceased trauma patients were treated by FinnHEMS per week. Low volume in critical care procedures in HEMS was also reported in a recently published study [31]. This is a challenge for maintenance of skills in pre-hospital trauma care. Nevertheless, HEMS providers likely gain more experience in triaging, treating, and transporting severely injured patients than EMS providers in ground ambulances operating in sparsely populated areas. This is supported by the fact that our patients were generally triaged to the correct level of care and on-scene time was acceptable (median, 18 min). Simulation-based training enables trauma team members to acquire and practice an array of tasks and to improve non-technical skills in trauma care [32]. Periodic work in trauma centers could compensate for the low number of pre-hospital trauma patients.

Helicopter transportation was used in only a minority of cases. Overutilization of HEMS for transportation was described in a previous meta-analysis [33]. However, this does not appear to be a problem in our region, as most patients were transported by ground ambulances and escorted by HEMS providers, if necessary. The low number of helicopter transportations may have resulted from the fact that therapeutic interventions are easier to perform in a ground ambulance. Landing sites located in-hospital and helicopters with larger cabins could change future practices.

The majority of patients were transported directly to the university hospital from the site of injury. In several cases, the nearest central hospital was passed according to the local protocol. Direct transportation of seriously injured patients from the site of injury to trauma centers has been shown to reduce mortality among trauma patients [9, 34] and this approach is reasonable for our region whenever possible.

This is the first study of FinnHEMS to describe outcomes and the trauma system in Northern Finland. Our results can be generalized to areas with long distances and sparse population. The national HEMS database was feasible for coupling pre-hospital data to patient records and the Cause of Death Registry in Finland, which is promising for future research purposes, such as prospective trauma outcome studies.

### Limitations

Our study has several limitations. First, the main limitation is the retrospective design. Second, some of the injured patients may have been classified as non-trauma patients in the HEMS database and not included in the study. Third, aborted trauma missions for which the reason was obvious trauma-related death were excluded. However, the aim of the study was to analyze trauma patients that survived to hospital, not to analyze all trauma patients in the area. Despite the retrospective design and the use of a number of different data sources, the amount of missing data was low, except for pre-hospital Glasgow Coma Score, blood pressure, on-scene and transport times. Lacking pre-hospital values made it difficult to use Trauma Injury Severity Score methodology; however, this methodology has limitations regarding the use of historical controls and a high misclassification rate [35, 36]. The on-scene and transport times are missing because of the fact that if the trauma patient is not escorted by FinnHEMS, the time when transport starts or the time of arrival at hospital are not routinely registered in the database.

The FinnHEMS units in our study were staffed by paramedics or anesthesiologists, which could be seen as a cofounding factor. However, our aim was to compare mortality between rural and urban trauma patients and to describe trauma care by FinnHEMS units, not to compare differences between types of HEMS units. It is important to acknowledge in this context that a physician-staffed HEMS is regularly consulted by a paramedic-staffed HEMS regarding logistics and treatment of a seriously injured patient.

Finally, because of the limited number of patients, we cannot rule out a type II error. Even though the difference in mortality for severely injured patients (ISS > 15) between the groups was not statistically significant, we believe that this difference was clinically important. Increasing the study population would have resulted in a longer time scale for the study, which could have negatively affected our results as therapies and treatments change over time.

## Conclusions

In conclusion, urban trauma patients who survived to hospital had a trend to higher 30-day mortality but with a similar length of ICU stay. A large proportion of deaths occurred in the pre-hospital setting before the arrival of HEMS.

## Abbreviations

EMS: emergency medical services
FinnHEMS: Finnish helicopter emergency medical services
HEMS: helicopter emergency medical services
ICU: intensive care unit
ISS: injury severity score

## References

[1] Statistics Finland (2014). Statistical Yearbook of Finland 2014.

[2] MacKenzie EJ, Rivara FP, Jurkovich GJ, Nathens AB, Frey KP, Egleston BL, Salkever DS, Scharfstein DO (2006). A national evaluation of the effect of trauma-center care on mortality. N Engl J Med.

[3] Celso B, Tepas J, Langland-Orban B, Pracht E, Papa L, Lottenberg L, Flint L (2006). A systematic review and meta-analysis comparing outcome of severely injured patients treated in trauma centers following the establishment of trauma systems. J Trauma.

[4] Kristiansen T, Soreide K, Ringdal KG, Rehn M, Kruger AJ, Reite A, Meling T, Nӕss PA, Lossius HM (2010). Trauma systems and early management of severe injuries in Scandinavia: review of the current state. Injury.

[5] Galvagno SM, Thomas S, Stephens C, Haut ER, Hirshon JM, Floccare D (2013). Helicopter emergency medical services for adults with major trauma. Cochrane Database Syst Rev.

[6] Krüger AJ, Lossius HM, Mikkelsen S, Kurola J, Castrén M, Skogvoll E (2013). Pre-hospital critical care by anaesthesiologist-staffed pre-hospital services in Scandinavia: a prospective population-based study. Acta Anaesthesiol Scand.

[7] Andruszkow H, Lefering R, Frink M, Mommsen P, Zeckey C, Rahe K, Krettek C, Hildebrand F (2013). Survival benefit of helicopter emergency medical services compared to ground emergency medical services in traumatized patients. Crit Care.

[8] Galvagno SM, Haut ER, Zafar SN, Millin MG, Efron DT, Koenig GJ, Baker SP, Bowman SM, Provonost PJ, Haider AH (2012). Association between helicopter vs ground emergency medical services and survival for adults with major trauma. JAMA.

[9] Desmettre T, Yeguiayan JM, Coadou H, Jacquot C, Raux M, Vivien B, Martin C, Bonithon-Kopp C, Freysz M, French Intensive Care Recorded in Severe Trauma (2012). Impact of emergency medical helicopter transport directly to a university hospital trauma center on mortality of severe blunt trauma patients until discharge. Crit Care.

[10] Bulger EM, Guffey D, Guyette FX, MacDonald RD, Brasel K, Kerby JD, Minei JP, Warden C, Rizoli S, Morrison LJ, Nichol G, Resuscitation Outcomes Consortium Investigators (2012). Impact of prehospital mode of transport after severe injury: a multicenter evaluation from the Resuscitation Outcomes Consortium. J Trauma Acute Care Surg.

[11] de Jongh MA, van Stel HF, Schrijvers AJ, Leenen LP, Verhofstad MH (2012). The effect of Helicopter Emergency Medical Services on trauma patient mortality in the Netherlands. Injury.

[12] Rose MK, Cummings GR, Rodning CB, Brevard SB, Gonzalez RP (2012). Is helicopter evacuation effective in rural trauma transport?. Am Surg.

[13] Bakke HK, Hansen IS, Bendixen AB, Morild I, Lilleng PK, Wisborg T (2013). Fatal injury as a function of rurality-a tale of two Norwegian counties. Scand J Trauma Resusc Emerg Med.

[14] Boland M, Staines A, Fitzpatrick P, Scallan E (2005). Urban–rural variation in mortality and hospital admission rates for unintentional injury in Ireland. Inj Prev.

[15] Fatovich DM, Jacobs IG (2009). The relationship between remoteness and trauma deaths in Western Australia. J Trauma.

[16] Kristiansen T, Lossius HM, Rehn M, Kristensen P, Gravseth HM, Røislien J, Søreide K (2014). Epidemiology of trauma: a population-based study of geographical risk factors for injury deaths in the working-age population of Norway. Injury.

[17] Harmsen AM, Giannakopoulos GF, Moerbeek PR, Jansma EP, Bonjer HJ, Bloemers FW (2015). The influence of prehospital time on trauma patients outcome: a systematic review. Injury.

[18] Lindström V, Pappinen J, Falk AC, Castrén M (2011). Implementation of a new emergency medical communication centre organization in Finland - an evaluation, with performance indicators. Scand J Trauma Resusc Emerg Med.

[19] Ringdal KG, Coats TJ, Lefering R, Di Bartolomeo S, Steen PA, Røise O, Handolin L, Lossius HM, Utstein TCD expert panel (2008). The Utstein template for uniform reporting of data following major trauma: a joint revision by SCANTEM, TARN, DGU-TR and RITG. Scand J Trauma Resusc Emerg Med.

[20] Baker SP, O´Neill B, Haddon W, Long WB (1974). The Injury Severity Score: a method for describing patients with multiple injuries and evaluating emergency care. J Trauma.

[21] Statistics Finland (2013). Handbooks. Municipalities and regional divisions based on municipalities.

[22] Gonzalez RP, Cummings GR, Phelan HA, Mulekar MS, Rodning CB (2009). Does increased emergency medical services prehospital time affect patient mortality in rural motor vehicle crashes? A statewide analysis. Am J Surg.

[23] Rogers FB, Shackford SR, Osler TM, Vane DW, Davis JH (1999). Rural trauma: the challenge for the next decade. J Trauma.

[24] McGuffie AC, Graham CA, Beard D, Henry JM, Fitzpatrick MO, Wilkie SC (2005). Scottish urban versus rural trauma outcome study. J Trauma.

[25] McCowan CL, Swanson ER, Thomas F, Handrahan DL (2007). Outcomes of blunt trauma victims transported by HEMS from rural and urban scenes. Prehosp Emerg Care.

[26] Fatovich DM, Phillips M, Jacobs IG, Langford SA (2011). Major trauma patients transferred from rural and remote Western Australia by the Royal Flying Doctor Service. J Trauma.

[27] Fatovich DM, Phillips M, Langfjord SA, Jacobs IG (2011). A comparison of metropolitan vs rural major trauma in Western Australia. Resuscitation.

[28] Kristiansen T, Lossius HM, Søreide K, Steen PA, Gaarder C, Næss PA (2011). Patients referred to a Norwegian Trauma Centre: effect of transfer distance on injury patterns, use of resources and outcomes. J Trauma Manag Outcomes.

[29] Rogers FB, Rittenhouse KJ, Gross BW (2015). The golden hour in trauma: dogma or medical folklore?. Injury.

[30] Hesselfeldt R, Steinmetz J, Jans H, Jacobsson ML, Andersen DL, Buggeskov K, Kowalski M, Præst M, Øllgaard L, Höiby P, Rasmussen LS (2013). Impact of a physician-staffed helicopter on a regional trauma system: a prospective, controlled, observational study. Acta Anaesthesiol Scand.

[31] Sollid SJ, Bredmose PP, Nakstad AR, Sandberg M (2015). A prospective survey of critical care procedures performed by physicians in helicopter emergency medical service: is clinical exposure enough to stay proficient?. Scand J Trauma Resusc Emerg Med.

[32] Gjeraa K, Møller TP, Østergaard D (2014). Efficacy of simulation-based trauma team training of non-technical skills. A systematic review. Acta Anaesthesiol Scand.

[33] Bledsoe BE, Wesley AK, Eckstein M, Dunn TM, O´Keefe MF (2006). Helicopter scene transport of trauma patients with nonlife-threatening injuries: a meta-analysis. J Trauma.

[34] Härtl R, Gerber LM, Ianoco L, Ni Q, Lyons K, Ghajar J (2006). Direct transport within an organized state trauma system reduces mortality in patients with severe traumatic brain injury. J Trauma.

[35] Galvagno SM (2013). Comparative effectiveness of helicopter emergency medical services compared to ground emergency medical services. Crit Care.

[36] Demetriades D, Chan L, Velmanos GV, Sava J, Preston C, Gruzinski G, Berne TV (2001). TRISS methodology: an inappropriate tool for comparing outcomes between trauma centers. J Am Coll Surg.

